# A Multi-Center, Prospective, Observational Study to Evaluate the Therapeutic Effectiveness and Safety of an Olmesartan/Amlodipine Plus Rosuvastatin Combination Treatment in Patients with Concomitant Hypertension and Dyslipidemia

**DOI:** 10.3390/jcm14020308

**Published:** 2025-01-07

**Authors:** Bong-Ki Lee, Byeong-Keuk Kim, Jae Hyoung Park, Jong-Won Chung, Chang Gyu Park, Jin Won Kim, Young Dae Kim, Woo-Jung Park, Sang-Hyun Kim, Jae-Kwan Cha, Cheol Ho Kim, Seung-Woon Rha, Young Joon Hong, Mi-Seung Shin, Seong Wook Cho, Young-Hee Sung, Kiheon Lee, Jae-Myung Yu, Dong-Ryeol Ryu, Sungwook Yu, Tae-Jin Song, Bon D Ku, Sin-Gon Kim, Hwan-Cheol Park, Deok-Kyu Cho, Byung-Su Kim, Seong-Woo Han, Sung-Ji Park, Gyung-Min Park, Kyoo-Rok Han

**Affiliations:** 1 Division of Cardiology, Department of Internal Medicine, Kangwon National University School of Medicine, Chuncheon 24289, Republic of Korea; mdbklee@kangwon.ac.kr (B.-K.L.); rdr0203@kangwon.ac.kr (D.-R.R.); 2 Department of Internal Medicine, Division of Cardiology, Yonsei University Severance Cardiovascular Hospital, Seoul 03722, Republic of Korea; kimbk@yuhs.ac; 3 Department of Internal Medicine, Division of Cardiology, Korea University Anam Hospital, Seoul 02841, Republic of Korea; jhpark3992@naver.com; 4 Departments of Neurology, Samsung Medical Center, Seoul 06351, Republic of Korea; neurocjw@gmail.com; 5 Departments of Neurology, Sungkyunkwan University School of Medicine, Seoul 06351, Republic of Korea; 6 Department of Internal Medicine, Division of Cardiology, Cardiovascular Center, Korea University Guro Hospital, Seoul 08308, Republic of Korea; parkcg@kumc.or.kr (C.G.P.); kjwmm@korea.ac.kr (J.W.K.); swrha617@yahoo.co.kr (S.-W.R.); 7 Department of Internal Medicine, Division of Cardiology, Dong-A University Hospital, Busan 49201, Republic of Korea; kimyd@dau.ac.kr; 8 Department of Internal Medicine, Division of Cardiovascular, Hallym University Sacred Heart Hospital, Anyang 14068, Republic of Korea; cathpark@hallym.or.kr; 9 Department of Internal Medicine, Division of Cardiology, Boramae Hospital, Seoul National University College of Medicine, Seoul 07061, Republic of Korea; shkimmd@snu.ac.kr; 10 Department of Internal Medicine, Division of Cardiology, Seoul National University College of Medicine, Seoul 07061, Republic of Korea; 11 Department of Neurology, Dong-A University Hospital, Busan 49201, Republic of Korea; nrcjk65@gmail.com; 12 Department of Internal Medicine, Cardiovascular Center, Seoul National University Bundang Hospital, Seongnam 13620, Republic of Korea; cheolkim55@gmail.com; 13 Department of Internal Medicine, Division of Cardiology, Chonnam National University Medical School, Gwangju 61469, Republic of Korea; hyj200@hanmail.net; 14 Department of Internal Medicine, Division of Cardiology, Gachon University Gil Medical Center, Incheon 21565, Republic of Korea; msshin@gilhospital.com; 15 Department of Internal Medicine, Division of Cardiology, Cardiovascular Medical Center, Bundang Jesaeng General Hospital, Seongnam 13590, Republic of Korea; cswim@dmc.or.kr; 16 Department of Neurology, Gachon University Gil Medical Center, Incheon 21565, Republic of Korea; atmann02@gilhospital.com; 17 Department of Family Medicine, Seoul National University Bundang Hospital, Seongnam 13620, Republic of Korea; keyhoney@gmail.com; 18 Department of Internal Medicine, Division of Endocrinology, Hallym University Kangnam Sacred Heart Hospital, Seoul 07441, Republic of Korea; jaemyungyu@hotmail.com; 19 Department of Neurology, Korea University Anam Hospital, Seoul 02841, Republic of Korea; song4yu@korea.ac.kr; 20 Department of Neurology, Seoul Hospital, Ewha Womans University, Seoul 07804, Republic of Korea; knstar@hanmail.net; 21 Department of Neurology, Catholic Kwandong University International ST. Mary’s Hospital, Incheon 22711, Republic of Korea; bondku@cku.ac.kr; 22 Department of Internal Medicine, Division of Endocrinology and Metabolism, Korea University Anam Hospital, Seoul 02841, Republic of Korea; k50367@korea.ac.kr; 23 Department of Internal Medicine, Division of Cardiology, Hanyang University Guri Hospital, Guri 11923, Republic of Korea; hcpark74@hanyang.ac.kr; 24 Department of Internal Medicine, Division of Cardiology, Yongin Severance Hospital, Yongin 16995, Republic of Korea; chodk123@yuhs.ac; 25 Department of Neurology, Ewha Womans University Mokdong Hospital, Seoul 07985, Republic of Korea; 01403@eumc.ac.kr; 26 Department of Neurology, Ewha Womans University College of Medicine, Seoul 07985, Republic of Korea; 27 Department of Internal Medicine, Division of Cardiology, Hallym University Dongtan Sacred Heart Hospital, Hwaseong 18450, Republic of Korea; hansw29@hallym.or.kr; 28 Department of Internal Medicine, Division of Cardiology, Samsung Medical Center, Seoul 06351, Republic of Korea; tyche.park@gmail.com; 29 Department of Internal Medicine, Division of Cardiology, Ulsan University Hospital, Ulsan 44033, Republic of Korea; min8684@hanmail.net; 30 Department of Internal Medicine, Division of Cardiology, Kangdong Sacred Heart Hospital, Seoul 05355, Republic of Korea; 31 Department of Internal Medicine, Division of Cardiology, Hallym University College of Medicine, Seoul 05355, Republic of Korea

**Keywords:** hypertension, dyslipidemia, blood pressure, low-density lipoprotein cholesterol, observational study, single-pill combination

## Abstract

**Introduction:** This study assessed the therapeutic effectiveness of a single-pill combination (SPC) of olmesartan/amlodipine plus rosuvastatin for blood pressure (BP) and low-density lipoprotein cholesterol (LDL-C) in patients with hypertension and dyslipidemia. **Methods:** Adult patients with hypertension and dyslipidemia who were decided to be treated with the study drug were eligible. The primary endpoint was the proportion of patients who achieved BP, LDL-C and both BP and LDL-C treatment goals at weeks 24–48. Secondary endpoints were assessed at weeks 24–48 and included changes in BP and LDL-C levels from baseline; the proportion of patients who achieved treatment goals who were initially classified as uncontrolled at baseline; changes and percent changes in lipid parameters; changes in both BP and LDL-C levels among patients who reached treatment goals who were followed for more than 24 weeks; and the overall safety profile. **Results:** A total of 5476 patients were enrolled, and 4411 patients comprised the effectiveness evaluation set. The proportions of patients who reached the treatment goals for BP, LDL-C levels, and both BP and LDL-C levels were 67.93% [95% confidence interval (CI) 66.52–69.32], 80.19% [95% CI 78.85–81.49], and 58.07% [95% CI 56.43–59.7], respectively. Secondary endpoints showed statistically significant changes. Overall, the treatment was well tolerated. **Conclusions:** The treatment of patients with hypertension and dyslipidemia with the olmesartan/amlodipine plus rosuvastatin SPC was associated with significant decreases in SBP/DBP and LDL-C levels, and a high proportion of patients achieved the BP and LDL-C treatment goals. The finding of this study is worthwhile in that this study evaluated the effectiveness and safety in a broad patient population representative of those seen in everyday clinical practice.

## 1. Introduction

Cardiovascular disease (CVD)-related mortality is a prominent contributor to global mortality rates, comprising approximately 30% of all deaths, as indicated by the World Health Organization’s 2021 report [1].

Epidemiological studies have demonstrated that an increase in cholesterol quintiles can lead to a threefold increased risk, while an increase in blood pressure (BP) quintiles can result in a three- to fourfold increased risk of CVD-related mortality. However, when these two risk factors are present concurrently, the risk may surge over tenfold, suggesting that the cardiovascular risk escalates exponentially [2]. On the contrary, the simultaneous use of non-pharmacological strategies and/or antihypertensive drugs and statins for managing hypertension and dyslipidemia leads to a significantly larger reduction in the risk of developing CVD [3]. To manage these multiple risk factors, numerous medications are necessary, which can lead to lower patient compliance [4]. To mitigate this problem, a single-pill combination (SPC) has been developed and implemented, showing improved adherence and effectiveness compared to free combination treatment [5,6,7]. In accordance with this, recent guidelines for hypertension proposed the commencement of SPC therapy for most patients on antihypertensive treatment. The favored combination involves renin–angiotensin–aldosterone system (RAAS) blockers along with calcium channel blockers (CCBs) [8]. Incorporating a statin into this antihypertensive regimen would form an optimal SPC for the concurrent control of hypertension and dyslipidemia. A combination of well-known drugs, olmesartan and amlodipine as antihypertensive medications and rosuvastatin as an antidyslipidemic drug, has demonstrated effectiveness and safety when compared to either olmesartan plus rosuvastatin or olmesartan plus amlodipine treatment in a previous phase 3 study [9].

In this observational study, we assessed the effectiveness and safety of an SPC containing olmesartan, amlodipine, and rosuvastatin (Olomax^®^, Daewoong Pharmaceutical Co., Ltd., Seoul, Republic of Korea) in patients with concomitant hypertension and dyslipidemia in real-world clinical setting, utilizing real-world data (RWD) and real-world evidence (RWE) gleaned from medical practice.

## 2. Methods

### 2.1. Purpose

This research, conducted from 27 December 2019, to 16 August 2022, was a prospective, observational study spanning 46 centers in Korea (NCT05184179). In this study, adult patients with hypertension and dyslipidemia were recruited to investigate the effectiveness and safety of Olomax in routine practice settings. The study protocol was approved by all the Institutional Review Boards, including Hallym University Sacred Heart Hospital (IRB No. KANGDONG 2020-03-002) and Kangwon National University Hospital (IRB No. KNUH-2019-12-006). At the time of enrollment, all patients provided written informed consent. This study was in alignment with the principles of the Declaration of Helsinki.

### 2.2. Study Population

Patients with concurrent hypertension and dyslipidemia aged ≥19 years and who were prescribed Olomax^®^ tablets based on physicians’ discretion were eligible. Those requiring three or more antihypertensive medications or two or more lipid-lowering medications were not included in the study. However, patients who had been already taking beta blockers (BBs) or diuretics for other conditions, such as angina or heart failure, that were not intended to treat hypertension were included. Patients who already had been taking Olomax^®^ and patients who were contraindicated to components of the study drug were deemed ineligible for the study. The exclusion criteria were (1) a history of hypersensitivity to olmesartan, rosuvastatin, or dihydropyridine-based derivatives; (2) pregnant and nursing women, and women of childbearing potential who were not using adequate contraception; (3) a severe hepatic impairment; (4) active liver disease, including persistent serum transaminase elevations of unknown cause or serum transaminase elevations greater than 3 times the upper normal limit; (5) biliary obstruction; (6) a severe renal impairment (creatinine clearance (CrCl) < 30 mL/min); (7) severe aortic stenosis; (8) myopathy; (9) concurrent cyclosporine administration; and (10) genetic issues such as galactose intolerance, Lapp lactase deficiency, or glucose–galactose malabsorption.

### 2.3. Study Definition

At enrollment, data on the participants’ demographics, past and concurrent medical problems, family history of cardiovascular disease (CVD), dates of diagnoses of hypertension and dyslipidemia, risk factors for coronary artery disease (CAD), and lifestyle factors (smoking and alcohol consumption) were gathered. Risk factors for coronary artery disease (CAD) were (1) smokers; (2) sitting systolic blood pressure (sitSBP) ≥140 mmHg or sitting diastolic blood pressure (sitDBP) ≥ 90 mmHg, or the use of antihypertensive medication; (3) a high-density lipoprotein cholesterol (HDL-C) level < 40 mg/dL, (4) ≥45 years for men and ≥55 years for women; and (5) a family history of early CAD. A family history of early CAD was defined as the occurrence of premature CAD in first-degree relatives (<55 or 65 years of age in men and women, respectively). When the HDL-C level was ≥60 mg/dL, one risk factor was subtracted from the total [10].

### 2.4. Study Medication

The study inclusively accepted patients with various therapeutic histories. Consequently, the study medication was prescribed as follows: (1) a first-line treatment for those previously untreated, (2) a substitute therapy for individuals already on other antihypertensive and lipid-lowering drugs, or (3) an additional treatment complementing a patient’s ongoing antihypertensive or lipid-lowering regimen. Patients were given a suitable daily dosage of the study medication. The study medication, composed of olmesartan medoxomil/amlodipine besylate/rosuvastatin calcium, was provided at one of four dosages, 20/5/5, 20/5/10, 40/5/5, or 40/5/10 mg, and was administered as a once daily treatment. Investigators had the clinical discretion to adjust the drug dosage either upward or downward as needed.

### 2.5. Effectiveness and Safety Assessments

The primary effectiveness measures were the percentage of patients achieving the 2018 Korean Society of Hypertension Guidelines sitSBP/sitDBP targets (for patients uncomplicated or aged ≥65 years, <140/90 mmHg; diabetics without CVD, <140/85 mmHg; and diabetics with CVD and at high risk [aged ≥ 50 years with CVD, peripheral artery disease (PAD), aortic disease, heart failure or left ventricular hypertrophy], <130/80 mmHg) and the Korean Guidelines for the Management of Dyslipidemia IV Low-density lipoprotein cholesterol (LDL-C) targets (for the very high-risk group, <70 mg/dL; high-risk group, <100 mg/dL; moderate-risk group, <130 mg/dL; and low-risk group, <160 mg/dL) at the end of the 24-week treatment period [11,12]. Secondary effectiveness measures included changes in baseline BP and LDL-C levels at weeks 24–48, the proportion of patients maintaining and achieving treatment goals, the changes in BP and LDL-C levels by the number of prior antihypertensive and antidyslipidemic agents, and the changes and rates of various lipid parameters [HDL-C, triglyceride (TG), and total cholesterol (TC), non-HDL]. Other secondary outcomes included the proportion of patients who reached the treatment goals for both BP and LDL-C levels among patients who were followed for 24 weeks or more, the amount of change from baseline, the change in the Framingham Risk Score (FRS) from baseline, the change in the carotid intima–media thickness (CIMT) from baseline, and the change in the high-sensitivity C-reactive protein (hsCRP) level from baseline, if data were available.

To evaluate the safety of the study drug, data for adverse events (AEs) and abnormal laboratory findings, either voluntarily reported by patients or identified by the treating physician during follow-up, were collected and assessed. AEs were categorized as treatment-emergent adverse events (TEAEs), adverse drug reactions (ADRs), serious adverse events (SAEs), and serious adverse drug reactions (SADRs).

### 2.6. Sample Size Estimation and Statistical Analysis

The number of patients in this study was calculated based on analyses of BP goal achievement and LDL-C goal achievement in the phase 3 study of Olomax^®^ [9]. Results from the phase 3 study showed that at 8 weeks of treatment with Olomax^®^, the rates of achievement of the BP goal and the LDL-C goal were approximately 84%. Due to the lack of literature on the effectiveness of the triple therapy beyond 24 weeks in patients with hypertension and dyslipidemia, we referenced a previous study on the effectiveness of amlodipine plus atorvastatin after 24 weeks and found that at 28 weeks, the proportion of patients who achieved the BP and LDL-C goals was 67.3% [13]. This means that at 24 weeks and beyond, the target achievement rate was expected to be 67.3–84%, and based on this, the meta-analysis confirmed that the effect size of the achievement rate was about 77%; so, we set the target BP and LDL-C achievement rates at 77% to calculate the number of patients.

The formula for calculating the sample size was as follows.

(1) $$n=\frac{{Z_{\alpha /2}}^{2}\times p\left(1-p\right)}{d^{2}}$$

- α: type 1 error (= 0.05);
- p: target achievement rate at week 24 and beyond (= 77%);
- *d*: margin of error (sampling error).

Thus, assuming an expected goal achievement rate of 77% at 24 weeks and beyond at a two-sided significance level of 0.05, and assuming that the margin of error would be in the range of 1% to 1.5%, setting the median value at 1.25% [i.e., the 95% confidence interval (CI) is 2.5% in length] yields a total number of 4355 study patients. Thus, to be 95% confident about the proportion of patients meeting the two primary endpoints of BP and LDL levels altogether between 75.75% and 78.25%, a total of 4355 patients would be needed. Factoring in a dropout rate of approximately 20%, a total of 5444 patients would be required, and so the study aimed to recruit an approximate final sample size of 5450 patients.

The effectiveness analysis set included patients who met the inclusion/exclusion criteria who participated in this observational study, were administrated Olomax^®^, and had effectiveness endpoints measured at week 24 and beyond. The safety analysis set included all patients in this study who were administrated Olomax^®^.

**Figure 1:**
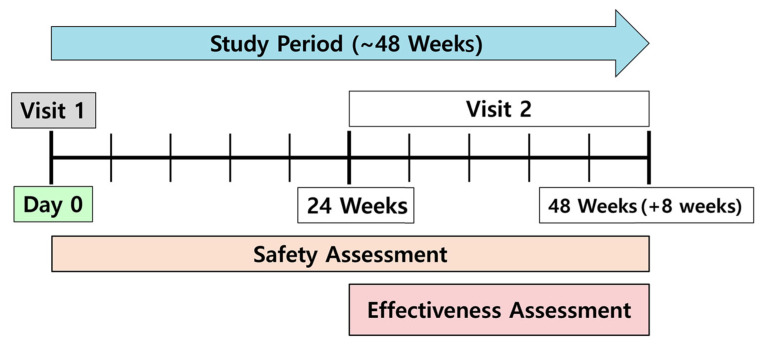
Schematic diagram of the study design.

Continuous variables are presented as the means and standard deviations (SDs), whereas categorical variables are presented as numbers and percentages. The exact Clopper–Pearson method was used to calculate 95% CI of the percentage of patients achieving the treatment goals at weeks 24–48. The significance of changes in blood pressure and lipid profiles from baseline to follow-up was tested using paired *t*-tests. All statistical analyses were performed using SAS software (version 8.2; SAS Institute, Inc., Cary, NC, USA) (Figure 1).

## 3. Results

### 3.1. Patient Disposition and Baseline Characteristics

A total of 5476 patients from 46 hospitals in Korea participated in this study. Of these patients, 83.02% completed the study within the time frame outlined in the protocol. Of the overall patients, 900 patients dropped out, and 30 patients were missing due to early termination of the study by the institution (Table 1). The safety set was 5401 patients, excluding 75 patients who were not administered the study drug. And 4411 patients were in the effectiveness evaluation set, excluding patients from the safety set for the following reasons: less than 24 weeks of study drug administration (817 patients), the effectiveness endpoint was not measured after study drug administration (814 patients), and ineligible for inclusion/exclusion criteria since the start of the study (196 patients).

The most commonly used single dose was 20/5/10 mg, accounting for 62.08% of patients, and the mean total administration duration of Olomax^®^ tablets was 227.64 days.

The mean age was 65.50 years; the proportion of male patients was 56.80%; and the mean body mass index (BMI) was 25.91 kg/m^2^. The mean duration of each indication was 9.03 years for hypertension and 5.87 years for dyslipidemia. The proportion of patients who received prior medication for hypertension and dyslipidemia before enrollment was 94.24%. None, monotherapy, a two-drug combination, and three or more drug combination of prior medications for hypertension were 3.56%, 22.24%, 69.16%, 4.17%, respectively, and none, monotherapy, and two or more drug combination of prior medications for dyslipidemia were 16.50%, 77.47%, 5.15%, respectively (Table 2).

### 3.2. Effectiveness Outcomes

#### 3.2.1. BP and LDL-C Treatment Goal Achievement

**Figure 2:**
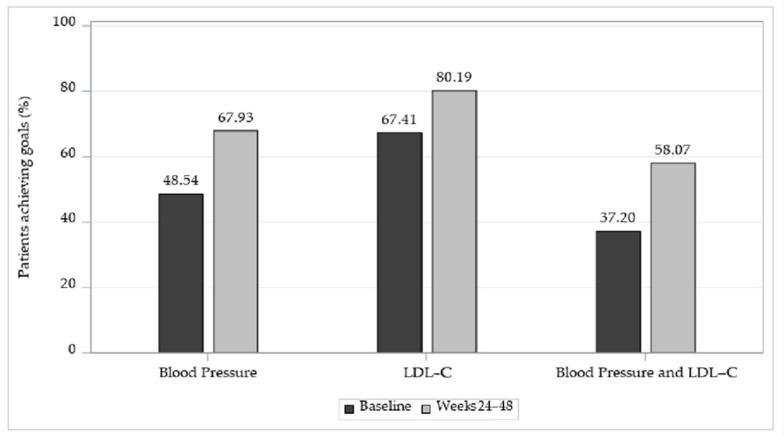
Proportions of patients with BP and LDL-C treatment goal achievement.

A total of 67.93% [95% CI 66.52–69.32] of patients achieved the treatment goal for BP (2949/4341 patients) and 80.19% [95% CI 78.85–81.49] of patients achieved the treatment goal for LDL-C levels (2879/3590 patients) at weeks 24–48. The proportion of patients who achieved the treatment goals for both BP and LDL-C levels at 24–48 weeks from baseline was 58.07% (2069/3563 patients) [95% CI 56.43–59.7] (Table 3 and Figure 2).

#### 3.2.2. Secondary Endpoints

**Figure 3:**
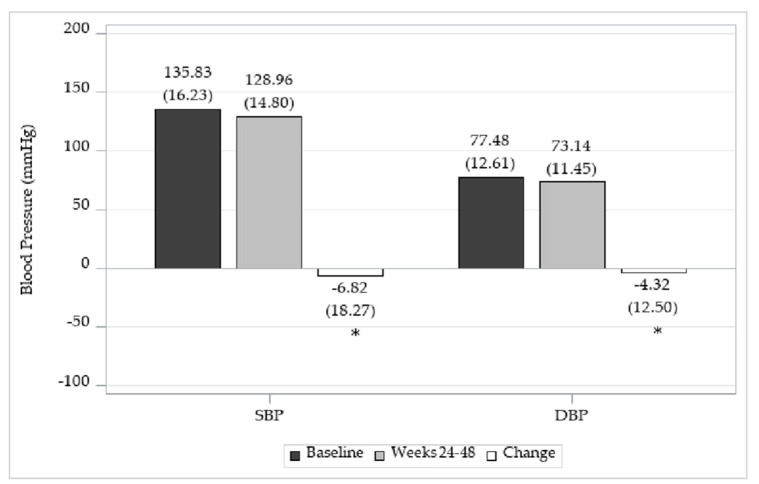
Change in BP. *: *p*-value < 0.05.

Baseline BP (mean ± SD) was 135.83 ± 16.23 for SBP and 77.48 ± 12.61 mmHg for DBP, and 128.96 ± 14.80 mmHg for SBP and 73.14 ± 11.45 mmHg for DBP at weeks 24–48. The BP was significantly reduced at weeks 24–48, with a change of −6.82 ± 18.27 mmHg for SBP (*p* < 0.0001) and −4.32 ± 12.50 mmHg for DBP (*p* < 0.0001) (Figure 3).

Among patients with uncontrolled BP at baseline, the baseline BP (mean ± SD) was 147.51 ± 12.16 mmHg for SBP and 83.32 ± 12.42 mmHg for DBP, and the BP was significantly reduced at weeks 24–48, with a change (mean ± SD) of −15.13 ± 17.55 mmHg for SBP (*p* < 0.0001) and −8.63 ± 12.61 mmHg for DBP (*p* < 0.0001). The proportion of patients who reached the treatment goal was 57.59% (1279/2221 patients) at weeks 24–48.

Patients with uncontrolled BP at baseline were categorized by the number of previous antihypertensive agents. For patients on monotherapy, the change (mean ± SD) in BP from baseline to weeks 24–48 was −21.07 ± 16.94 mmHg for SBP (*p* < 0.0001) and −11.86 ± 11.98 mmHg for DBP (*p* < 0.0001). The proportion of patients with treatment target achievement at weeks 24–48 was 64.74% (415/641 patients). For patients on two-drug combination therapy, the change (mean ± SD) in BP from baseline to weeks 24–48 was −10.99 ± 15.69 mmHg for SBP (*p* < 0.0001) and −5.84 ± 11.58 mmHg for DBP (*p* < 0.0001). The proportion of patients with treatment target achievement at week 24–48 was 53.72% (678/1262 patients). For patients on three or more drug combination therapy, the change (mean ± SD) in BP from baseline to weeks 24–48 was −6.11 ± 17.66 mmHg for SBP (*p* = 0.0084) and −5.10 ± 13.92 mmHg for DBP (*p* = 0.0038). The proportion of patients with treatment target achievement at weeks 24–48 was 38.71% (24/62 patients).

The baseline LDL-C level (mean ± SD) was 89.05 ± 33.25 mg/dL, and 74.80 ± 22.55 mg/dL at weeks 24–48. The LDL-C level was significantly reduced at weeks 24–48, with a change (mean ± SD) of −13.46 ± 31.69 mg/dL (*p* < 0.0001), and the percent change (mean ± SD) from baseline to weeks 24–48 was −6.96 ± 55.06%.

Among patients with uncontrolled LDL-C levels at baseline, the baseline LDL-C level (mean ± SD) was 110.41 ± 37.24 mg/dL, the LDL-C level was significantly reduced at weeks 24–48, with a change (mean ± SD) of −29.17 ± 37.57 mg/dL (*p* < 0.0001), and the percent change (mean ± SD) from baseline to weeks 24–48 was −19.67 ± 30.36%. The proportion of patients who reached the treatment goal was 61.31% (672/1096 patients) at weeks 24–48.

Patients with uncontrolled LDL-C levels at baseline were categorized by the number of previous antidyslipidemic agents. For patients who had not been given therapy, the change (mean ± SD) in LDL-C levels from baseline to weeks 24–48 was −59.41 ± 32.92 mg/dL (*p* < 0.0001) and the percent change (mean ± SD) from baseline to weeks 24–48 was −40.12 ± 21.54%. The proportion of patients with treatment target achievement at weeks 24–48 was 78.91% (202/256 patients). For patients on monotherapy, the change (mean ± SD) in LDL-C levels from baseline to weeks 24–48 was −16.11 ± 29.65 mg/dL (*p* < 0.0001) and the percent change (mean ± SD) was −11.62 ± 28.60%. The proportion of patients with treatment target achievement at weeks 24–48 was 54.74% (381/696 patients). For patients on two or more drug combination therapy, the change (mean ± SD) in LDL-C levels from baseline to weeks 24–48 was 5.13 ± 25.17 mg/dL; however, the result was not statistically significant (*p* = 0.1838). The percent change (mean ± SD) from baseline was 11.83 ± 29.17%. The proportion of patients with treatment target achievement at weeks 24–48 was 43.18% (19/25 patients).

**Figure 4:**
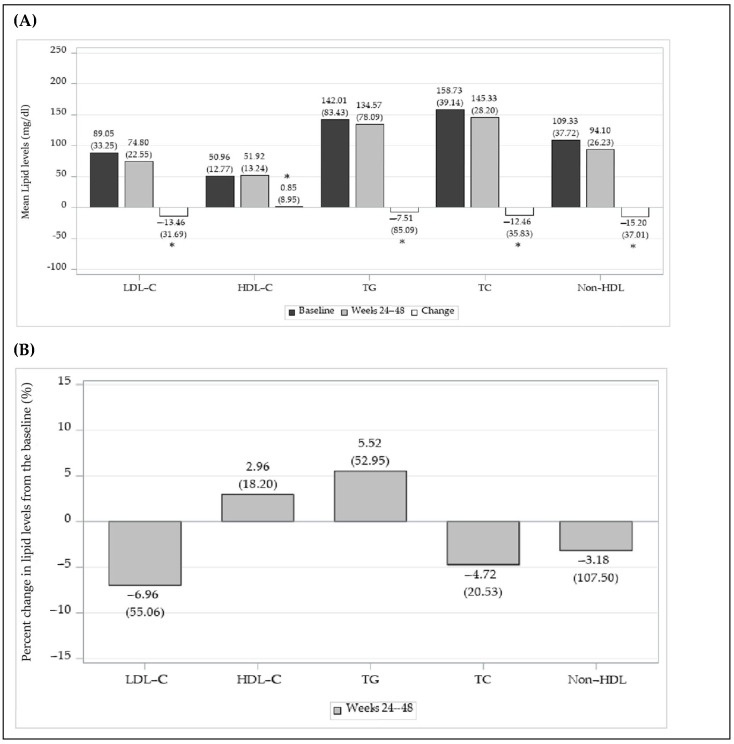
Changes in lipid parameters in the effectiveness evaluation set. (**A**) Changes in LDL-C, HDL-C, triglyceride, total cholesterol, non-HDL-cholesterol levels between the baseline and weeks 24–48 *: *p*-value < 0.05. (**B**) Percent changes in LDL-C, HDL-C, TG, TC, and non-HDL levels from the baseline.

The change in HDL-C levels was significantly increased, and the changes in TG, TC and non-HDL levels were significantly reduced at weeks 24–48 (*p* < 0.0001). The percent changes in lipid parameters except TG were similar to each change, but the percent change in TG levels increased (Figure 4).

**Figure 5:**
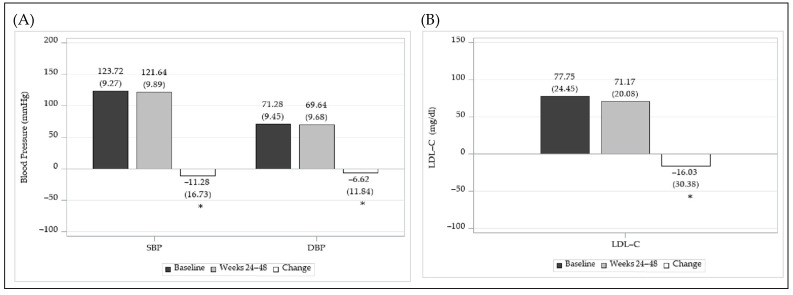
Changes in the BP and LDL-C levels of patients who were followed for more 24 weeks in the effectiveness evaluation set. (**A**) Levels at baseline and weeks 24–48 and changes in BP. *: *p*-value < 0.05. (**B**) Levels at baseline and weeks 24–48 and changes in LDL-C levels. *: *p*-value < 0.05.

Among the patients who reached the treatment goals for both BP and LDL-C levels at weeks 24–48, changes from the baseline (means ± SDs) were −11.28 ± 16.73 mmHg for SBP, −6.62 ± 11.84 mmHg for DBP, and −16.03 ± 30.38 mg/dL for LDL-C levels (all *p* < 0.0001) (Figure 5).

The additional results are reported in the Supplementary Materials.

### 3.3. Safety Outcomes

The overall adverse event rate was 15.11% (816/5401) and the total adverse drug reaction (ADR) rate was 3.24% (175/5401). No single adverse event occurred at a rate greater than 3%. Regarding the severity of adverse drug reactions, ADRs, mild cases were the most common, and moderate ADRs occurred in 0.37% of patients (Table 4).

‘Dizziness’ was the most common adverse event, which occurred in 2.35% (129 cases in 127 patients). It was the most common adverse drug reaction (1.17%, 63 cases in 63 patients) and the most common adverse event leading to drug discontinuation (0.93%, 50 cases in 50 patients) as well. Other frequent adverse events reported in order were ‘headache’, ‘chest pain’, and ‘dyspnea’. Also, adverse drug reactions were reported in the order of ‘hypotension’, ‘headache’, and ‘hypertension’. Instances of edema, tachycardia, facial flushing, and muscle pain were infrequent and below 1% each. ‘Cardiac arrest’ and ‘hepatitis’ were two reported serious adverse drug reactions (SADRs). Both SADRs were recovered AEs, and the study drugs were discontinued after occurrence. The main cause of the cardiac arrest was considered hyperkalemia, and it was judged that the study drug might have affected the electrolyte abnormality. However, since there were other causes of hyperkalemia, including underlying diseases and concomitant medications such as psychiatric drugs, the causal relationship between the cardiac arrest and the study drug could not be established.

## 4. Discussion

In this observational study, we assessed the long-term effectiveness and safety of an SPC of olmesartan, amlodipine and rosuvastatin in patients concurrently presenting with hypertension and dyslipidemia. Olomax^®^ is an SPC that received marketing authorization in February 2019 from the Ministry of Food and Drug Safety in South Korea. Previous clinical trials have demonstrated significant benefits in controlling hypertension and dyslipidemia with the combination therapy [9,14]. The study drug proficiently met the BP and LDL-C objectives and exhibited a favorable safety profile without any novel adverse observations in a real-world clinical setting.

ARBs and CCBs are the first-line medications used to control BP effectively [15]. Furthermore, given that the two agents have different mechanisms of action, recent guidelines recommend that the initial SPC therapy be applied in patients with stage 2 hypertension or SBP/DBP who require a reduction of at least 20/10 mmHg [11,16,17,18]. Rosuvastatin, an 3-hydroxy-3-methylglutaryl coenzyme A reductase inhibitor, is a widely used LDL-C-lowering drug. Several studies have demonstrated the benefits of treating hypertension and dyslipidemia with a combination of antihypertensives and statins, including a significant reduction in the cardiovascular risk compared to management with separate medicines [3,19].

In this study, the proportion of patients who achieved the treatment goals for both BP and LDL-C levels at weeks 24–48 was 58.07%, and the result is lower than the expected result of 77%. This discrepancy could be attributed to the fact that the predicted value was derived from clinical trials rather than RWD, highlighting a gap between clinical trials and observational studies. Several factors may contribute to this difference, including inadequate patient follow-up, the absence of well-defined protocols, limited adherence controls, cost considerations, and a lack of patient motivation. Additionally, the exclusion of study populations with severe complications in clinical trials using higher dosages, as well as repeated BP measurements, could further widen the observed gap. However, when compared to other observational studies, the achievement rates in this study were found to be similar [20,21,22,23].

In addition to their known effects on serum LDL-C levels, statin drugs have been demonstrated to affect triglyceride levels minimally [24]. This study found a statistically significant reduction in TG levels from baseline, with all other lipid parameters also significantly altered. However, the percent change in TG levels showed an increasing pattern, which was due to the very low or high, and rapidly changing data. Since the fasting state was not evenly controlled before the lipid panel measurement and TG is a variable that is more affected by diet than other lipid parameters, there seemed to be a lot of interpersonal variabilities and intrapersonal variabilities between each visit. The extent of changes in BP and LDL-C levels varied according to the number of prior medications. Among patients with a previous antihypertensive medication history, those on monotherapy showed greater reductions in BP than those on two-drug combination therapy. Similarly, patients without prior antidyslipidemic medication also showed greater reductions in LDL-C levels compared to those on monotherapy.

The 2024 European Society of Cardiology (ESC) guidelines for the management of elevated blood pressure and hypertension identified poor adherence to treatment and physicians’ clinical inertia (i.e., a lack of therapeutic action when a patient’s BP is uncontrolled) as significant causes of BP control failure [16]. Furthermore, the relationship between poor adherence to treatment and a higher cardiovascular disease risk has been widely reported [25]. Of note, 57.59% of patients with uncontrolled BP at baseline achieved the treatment goal after administration of the study drug at weeks 24–48. Also, more than 60% of patients with uncontrolled LDL-C levels at baseline achieved the treatment goal at weeks 24–48. We believe that this promising result came from the well-known fact that SPC therapy is an effective method for enhancing adherence.

The findings of this study are meaningful in many ways. First, this study included a considerable number of patients with various underlying diseases. Next, in contrast to previous studies, this study used a RWD in a real-world practice setting. Lastly, the follow-up period was up to 48 weeks, which is longer than the previous studies, allowing us to confirm the long-term effects of Olomax^®^.

### Limitations

This study has several limitations. As stated above, potential confounding variables, such as lifestyle modifications, were not controlled in this study due to its observational nature. Second, although there were clear correlations between the administration of Olomax^®^ and reductions in both BP and LDL-C levels, this study cannot prove causality as an observational study. Third, the BP measurement method was not standardized in this study, and there may be variations due to the influence of external factors, as there were differences in the repeated measurements of BP and the resting state before measurement. Fourth, lipid parameter data for some patients were not documented, and so the results of data regarding lipid parameters might not represent the whole study population. Fifth, myocardial overload parameters such as Troponin T and NT-proBNP were not included in the protocol of this observational study, as it primarily focused on blood pressure and lipid parameters. Lastly, the study period overlapped with the COVID-19 pandemic. While we did not specifically record the COVID-19 infection status as part of the protocol, we have reviewed the clinical data and found no direct indications that a COVID-19-related illness affected the study outcomes or participant safety.

## 5. Conclusions

The single-pill combination of olmesartan/amlodipine plus rosuvastatin was significantly associated with reductions in BP and LDL-C levels to the treatment goals, with an excellent safety profile, regardless of the patient’s previous medication regimen.

## Figures and Tables

**Table 1:** Participation information and degree of exposure.

	Overall Patients (n = 5476)
Completed, n (%)	4546 (83.02)
Dropped out, n (%)	900 (16.44)
Missing, n (%)	30 (00.54)
Total duration of prescription (days), mean (SD)	242.52 (109.97)
Total duration of administration (days), mean (SD)	227.64 (96.20)

SD: standard deviation.

**Table 2:** Baseline characteristics.

Characteristic	Safety Set (n = 5401)
Age (years), mean (SD)	65.50 (11.70)
Sex, n (%)	
Male	3068 (56.80)
Female	2333 (43.20)
BMI (kg/m^2^), mean (SD) ^1^	25.91 (3.61)
Duration of disease (years), mean (SD) ^2^	
Hypertension	9.03 (7.99)
Dyslipidemia	5.87 (5.93)
Major risk factors for coronary artery disease, n (%) ^3^	
Male ≥45 years of age or female ≥55 years of age Diabetes mellitus	4959 (92.17) 1764 (32.67)
Smoking	803 (14.93)
BP ≥140/90 mmHg or higher or taking antihypertensive medication	5030 (93.49)
HDL < 40 mg/dL	886 (16.47)
Family history of premature coronary artery disease	43 (0.80)
Number of major risk factors for coronary artery disease, mean (SD) ^2^	2.17 (0.66)
Number of prior medications for hypertension, n (%) ^4^	
None	181 (3.56)
Monotherapy	1132 (22.24)
Two-drug combination	3520 (69.16)
Three or more drug combination	212 (4.17)
Number of prior medications for dyslipidemia, n (%) ^4^	
None	840 (16.50)
Monotherapy	3943 (77.47)
Two or more drug combination	262 (5.15)

SD: standard deviation, HDL: high-density lipoprotein–cholesterol. ^1^ The number of patients is 3661. ^2^ The number of patients is 5400. ^3^ The number of patients is 5380. ^4^ The number of patients is 5090. The number with unknown prior hypertension and dyslipidemia medications is 45 patients.

**Table 3:** Proportion of patients with BP and LDL-C treatment goal achievement.

**Patient Number (%)**	**Effectiveness Evaluation Set** **(n = 4411)**	
	**n (%)**	**95% CI**
BP treatment goal achievement ^1^	2949 (67.93)	[66.52, 69.32]
LDL-C treatment goal achievement ^2^	2879 (80.19)	[78.85, 81.49]
BP and LDL-C treatment goal achievement ^3^	2069 (58.07)	[56.43, 59.7]

CI: confidence interval, BP: blood pressure, LDL-C: low-density lipoprotein–cholesterol. ^1^ The number of patients is 4341. ^2^ The number of patients is 3590. ^3^ The number of patients is 3563.

**Table 4:** Summary of adverse events in the safety set.

Characteristic	Safety Set (n = 5401)
**TEAEs, n (%)**	816 (15.11)
**ADRs, n (%)**	175 (3.24)
**Severity**	
Mild	155 (2.87)
Moderate	20 (0.37)
Severe	0 (0)
**SAEs, n (%)**	105 (1.94)
**SADRs, n (%)**	2 (0.04)
**TEAEs Leading to Drug Discontinuations, n (%)**	194 (3.59)

TEAEs = treatment-emergent adverse events; ADRs = adverse drug reactions; SAEs = serious adverse events, SADRs = serious adverse drug reactions.

## Data Availability

Data are contained within the article and Supplementary Materials.

## Supplementary Materials

The following supporting information can be downloaded at https://www.mdpi.com/article/10.3390/jcm14020308/s1: Table S1. BP change and treatment goal retention rate from baseline to weeks 24–48 among patients whose BP was already controlled at baseline; Table S2. BP change and treatment goal retention rate by number of prior antihypertensive agents from baseline to weeks 24–48 among patients whose BP was already controlled at baseline; Table S3. LDL-C change and treatment goal retention rate from baseline to weeks 24–48 among patients whose LDL-C levels were already controlled at baseline; Table S4. LDL-C change, % change and treatment goal retention rate by number of prior antidyslipidemic agents from baseline to weeks 24–48 among patients whose LDL-C levels were already controlled at baseline; Table S5. Change in the Framingham Risk Score (FRS) from baseline; Table S6. Change in the carotid intima–media thickness (CIMT) from baseline; Table S7. Change in hsCRP levels from baseline.
