# Supplementary material for: A Multi-Center, Prospective, Observational Study to Evaluate the Therapeutic Effectiveness and Safety of an Olmesartan/Amlodipine Plus Rosuvastatin Combination Treatment in Patients with Concomitant Hypertension and Dyslipidemia
Source: J Clin Med. 2025 Jan 7;14(2):308. doi: 10.3390/jcm14020308 (PMC11765630; doi:10.3390/jcm14020308)
Supplement: Supplementary file 1 [file jcm-14-00308-s001.zip › jcm-3384462-supplementary.pdf]

Table S1 BP change and treatment goal retention rate from baseline to week 24-48 among patients with BP already controlled at baseline

	Effectiveness evaluation Set (N=4411)	
	Result	Change from Baseline
**SBP (mmHg)**		
Baseline		
Number of subjects	2129	
Mean	123.44	
Standard deviation	9.37	
Median	124.00	
Minimum	80.00	
Maximum	149.00	
Week 24-48		
Number of subjects	2099	2099
Mean	125.43	1.97
Standard deviation	13.42	14.53
Median	125.00	0.00
Minimum	80.00	-40.00
Maximum	207.00	72.00
p-value[1]		<0.0001*
**DBP (mmHg)**		
Baseline		
Number of subjects	2129	
Mean	71.28	
Standard deviation	9.47	
Median	72.00	
Minimum	35.00	
Maximum	89.00	
Week 24-48		

	Effectiveness evaluation Set (N=4411)	
	**Result**	**Change from Baseline**
Number of subjects	2098	2098
Mean	71.51	0.23
Standard deviation	10.78	10.63
Median	72.00	0.00
Minimum	34.00	-38.00
Maximum	111.00	52.00
p-value[1]		0.8653

Treatment goal retention rate

Week 24-48		
Number of subjects	2098	
Achieve	1655 (78.88)	
Fail	443 (21.12)	

[1]Paired t-test or Wilcoxon's signed rank sum test * Statistically significant at the 5% significance level on both sides.

Table S2 BP change and treatment goal retention rate by number of prior antihypertensive agents from baseline to week 24-48 among patients with BP already controlled at baseline

	Effectiveness evaluation Set (N=4411)	
	**Result**	**Change from Baseline**
**Monotherapy**		
**SBP (mmHg)**		
Baseline		
Number of subjects	265	
Mean	125.31	
Standard deviation	9.24	
Median	126.00	
Minimum	82.00	
Maximum	139.00	
Week 24-48		
Number of subjects	262	262
Mean	122.39	-2.80
Standard deviation	12.80	14.63
Median	121.50	-4.00
Minimum	89.00	-40.00
Maximum	207.00	72.00
p-value[1]		0.0002*
**DBP (mmHg)**		
Baseline		
Number of subjects	265	
Mean	73.52	
Standard deviation	8.68	
Median	75.00	
Minimum	46.00	
Maximum	89.00	

	Effectiveness evaluation Set (N=4411)	
	Result	Change from Baseline
Week 24-48		
Number of subjects	262	262
Mean	70.40	-3.12
Standard deviation	9.42	9.99
Median	71.00	-3.00
Minimum	41.00	-35.00
Maximum	98.00	28.00
p-value[1]		<0.0001*
**Treatment goal retention rate**		
Week 24-48		
Number of subjects	262	
Achieve	221 (84.35)	
Fail	41 (15.65)	
**Two-drug combination**		
**SBP (mmHg)**		
Baseline		
Number of subjects	1685	
Mean	123.38	
Standard deviation	9.21	
Median	124.00	
Minimum	82.00	
Maximum	149.00	
Week 24-48		
Number of subjects	1662	1662
Mean	125.64	2.22
Standard deviation	13.38	14.17

	Effectiveness evaluation Set (N=4411)	
	Result	Change from Baseline
Median	125.00	1.00
Minimum	80.00	-39.00
Maximum	177.00	57.00
p-value[1]		<0.0001*
**DBP (mmHg)**		
Baseline		
Number of subjects	1685	
Mean	71.16	
Standard deviation	9.50	
Median	72.00	
Minimum	35.00	
Maximum	89.00	
Week 24-48		
Number of subjects	1661	1661
Mean	71.62	0.48
Standard deviation	10.94	10.49
Median	72.00	0.00
Minimum	34.00	-38.00
Maximum	111.00	52.00
p-value[1]		0.2905
**Treatment goal retention rate**		
Week 24-48		
Number of subjects	1661	
Achieve	1305 (78.57)	
Fail	356 (21.43)	
**Three or more drug combination**		

	Effectiveness evaluation Set (N=4411)	
	Result	Change from Baseline
**SBP (mmHg)**		
Baseline		
Number of subjects	88	
Mean	120.49	
Standard deviation	11.15	
Median	122.00	
Minimum	80.00	
Maximum	139.00	
Week 24-48		
Number of subjects	87	87
Mean	130.37	9.99
Standard deviation	13.92	14.68
Median	130.00	9.00
Minimum	98.00	-23.00
Maximum	169.00	51.00
p-value[1]		<0.0001*
**DBP (mmHg)**		
Baseline		
Number of subjects	88	
Mean	67.27	
Standard deviation	10.08	
Median	67.50	
Minimum	44.00	
Maximum	89.00	
Week 24-48		
Number of subjects	87	87
Mean	73.64	6.52
Standard deviation	11.84	11.05

	Effectiveness evaluation Set (N=4411)	
	Result	Change from Baseline
Median	73.00	6.00
Minimum	39.00	-18.00
Maximum	105.00	39.00
p-value[1]		<0.0001*

Treatment goal retention rate

Week 24-48		
Number of subjects	87	
Achieve	59 (67.82)	
Fail	28 (32.18)	

[1]Paired t-test or Wilcoxon's signed rank sum test * Statistically significant at the 5% significance level on both sides.

Table S3 LDL-C change, % change and treatment goal retention rate from baseline to week 24-48 among patients with LDL-C already controlled at baseline

	Effectiveness evaluation Set (N=4411)		
(mg/dl)	Result	Change from Baseline	% Change from Baseline
Baseline			
Number of subjects	2700		
Mean	78.82		
Standard deviation	25.29		
Median	76.00		
Minimum	9.40		
Maximum	239.00		
Week 24-48			
Number of subjects	2258	2257	2257
Mean	72.46	-5.92	-2.00
Standard deviation	21.97	24.93	33.67
Median	70.00	-4.00	-4.94
Minimum	3.60	-131.00	-95.50
Maximum	231.00	124.60	296.67
p-value[1]		<0.0001*	
**Treatment goal retention rate**			
Week 24-48			
Number of subjects	2255		
Achieve	2018 (89.49)		
Fail	237 (10.51)		

[1]Paired t-test or Wilcoxon's signed rank sum test * Statistically significant at the 5% significance level on both sides.

Table S4 LDL-C change, % change and treatment goal retention rate by number of prior antidyslipidemic agents from baseline to week 24-48 among patients with LDL-C already controlled at baseline

	Effectiveness evaluation Set (N=4411)		
**(mg/dl)**	**Result**	**Change from Baseline**	**% Change from Baseline**
**None**			
Baseline			
Number of subjects	299		
Mean	95.82		
Standard deviation	34.64		
Median	94.00		
Minimum	26.00		
Maximum	210.00		
Week 24-48			
Number of subjects	231	231	231
Mean	69.16	-25.35	-17.61
Standard deviation	22.28	35.90	36.74
Median	68.00	-22.00	-25.23
Minimum	3.60	-131.00	-95.50
Maximum	166.00	44.40	119.23
p-value[1]		<0.0001*	
**Treatment goal retention rate**			
Week 24-48			
Number of subjects	229		
Achieve	207 (90.39)		
Fail	22 (9.61)		
**Monotherapy**			

	Effectiveness evaluation Set (N=4411)		
(mg/dl)	Result	Change from Baseline	% Change from Baseline
Baseline			
Number of subjects	2212		
Mean	76.15		
Standard deviation	21.85		
Median	75.00		
Minimum	9.40		
Maximum	239.00		
Week 24-48			
Number of subjects	1871	1870	1870
Mean	72.30	-3.59	-0.74
Standard deviation	21.28	20.25	31.13
Median	70.00	-3.00	-4.32
Minimum	12.60	-103.60	-78.57
Maximum	166.60	124.60	296.67
p-value[1]		<0.0001*	
**Treatment goal retention rate**			
Week 24-48			
Number of patients	1870		
Achieve	1687 (90.21)		
Fail	183 (9.79)		
**Two-drug combination**			
Baseline			
Number of subjects	103		
Mean	68.83		
Standard deviation	21.03		
Median	66.00		
Minimum	29.00		

(mg/dl)	Effectiveness evaluation Set (N=4411) Result	Change from Baseline	% Change from Baseline
Maximum	124.00		
Week 24-48			
Number of subjects	87	87	87
Mean	82.27	12.58	25.90
Standard deviation	26.13	29.08	50.12
Median	79.00	9.00	12.75
Minimum	40.00	-79.00	-66.39
Maximum	169.00	118.00	295.00
p-value[1]		<0.0001*	
**Treatment goal retention rate**			
Week 24-48			
Number of patients	87		
Achieve	69 (79.31)		
Fail	18 (20.69)		

[1]Paired t-test or Wilcoxon's signed rank sum test * Statistically significant at the 5% significance level on both sides.

Table S5 Change in Framingham Risk Score (FRS) from baseline

	Effectiveness evaluation Set (N=4411)	
	Result	Change from Baseline
**Risk points**		
Baseline		
Number of subjects	2482	
Mean	70.49	
Standard deviation	18.57	
Median	76.00	
Minimum	0.50	
Maximum	86.00	
Week 24-48		
Number of subjects	2107	1956
Mean	67.67	-2.89
Standard deviation	18.03	15.53
Median	71.00	0.00
Minimum	0.17	-76.80
Maximum	85.00	83.00
p-value[1]		<0.0001*
**10-year risk in %**		
Baseline		
Number of subjects	2482	
Mean	20.21	
Standard deviation	12.29	
Median	18.80	
Minimum	0.80	
Maximum	85.00	
Week 24-48		
Number of subjects	2107	1956

	Effectiveness evaluation Set (N=4411)	
	Result	Change from Baseline
Mean	17.42	-2.59
Standard deviation	11.60	12.88
Median	15.00	-0.85
Minimum	0.30	-76.70
Maximum	120.00	100.40
p-value[1]		<0.0001*

[1]Paired t-test or Wilcoxon's signed rank sum test * Statistically significant at the 5% significance level on both sides.

Table S6 Change in carotid intima-media thickness (CIMT) from baseline

	Effectiveness evaluation Set (N=4411)	
**(mm)**	**Result**	**Change from Baseline**
Baseline		
Number of subjects	145	
Mean	1.65	
Standard deviation	2.29	
Median	0.90	
Minimum	0.50	
Maximum	12.90	
Week 24-48		
Number of subjects	34	24
Mean	1.13	0.00
Standard deviation	0.59	0.15
Median	1.00	0.00
Minimum	0.60	-0.51
Maximum	4.19	0.42
p-value[1]		1.0000

[1]Paired t-test or Wilcoxon's signed rank sum test

Table S7 Change in hsCRP from baseline

	Effectiveness evaluation Set (N=4411)	
**(mg/dl)**	**Result**	**Change from Baseline**
Baseline		
Number of subjects	780	
Mean	0.51	
Standard deviation	1.81	
Median	0.11	
Minimum	0.01	
Maximum	37.28	
Week 24-48		
Number of subjects	556	352
Mean	0.52	0.01
Standard deviation	1.58	1.10
Median	0.10	0.00
Minimum	0.01	-9.09
Maximum	17.48	9.94
p-value[1]		0.8226

[1]Paired t-test or Wilcoxon's signed rank sum test
